# Ferrostatin-1 ameliorates Cis-dichlorodiammineplatinum(II)-induced ovarian toxicity by inhibiting ferroptosis

**DOI:** 10.1186/s10020-024-00923-7

**Published:** 2024-09-13

**Authors:** Lu Zhang, Zhe Dong, Fan Jiang, Huaju Huang, Hui Ding, Meimei Liu

**Affiliations:** 1 Harbin Medical University, Harbin, 150086 Heilongjiang China; 2 Department of Obstetrics and Gynecology, The Second Affiliated Hospital of Harbin Medical University, Harbin, 150086 Heilongjiang China; 3 Jinzhou Medical University, Jinzhou, 121000 Liaoning China

**Keywords:** Cis-dichlorodiammine platinum (II), CDDP, Ovarian toxicity, Ferroptosis, FER-1

## Abstract

Cis-dichlorodiammineplatinum(II) (CDDP), while widely utilized in tumor therapy, results in toxic side effects that patients find intolerable. The specific mechanism by which CDDP inflicts ovarian damage remains unclear. This study aimed to explore the involvement of ferrostatin-1 (FER-1) and ferroptosis in CDDP-induced ovarian toxicity. This study established models of CDDP-induced injury in granulosa cells (GCs) and rat model of premature ovarian failure (POF). CCK-8 assessed the effects of CDDP and FER-1 on GC viability. FerroOrange and Mito-FerroGreen, DCFH-DA and MitoSox-Red, Rhodamine 123 and Transmission electron microscopy (TEM) measured Fe^2+^, reactive oxygen species (ROS), mitochondrial membrane potential and the mitochondrial morphology in GC cells, respectively. Serum hormone levels; organ indices; malondialdehyde, superoxide dismutase, and glutathione analyses; and western blotting were performed to examine ferroptosis's role in vitro. Molecular docking simulation was evaluated the interaction between FER-1 and GPX4 or FER-1 and NRF2. Molecular docking simulations were conducted to evaluate the interactions between FER-1 and GPX4, as well as FER-1 and NRF2. The findings revealed that CDDP-induced ovarian toxicity involved iron accumulation, increased ROS accumulation, and mitochondrial dysfunction, leading to endocrine disruption and tissue damage in rats. These changes correlated with NRF2, HO-1, and GPX4 levels. However, FER-1 decreased the extent of ferroptosis. Thus, ferroptosis appears to be a crucial mechanism of CDDP-induced ovarian injury, with GPX4 as potential protective targets.

**Supplementary Information:**

The online version contains supplementary material available at 10.1186/s10020-024-00923-7.

## Introduction

Cis-dichlorodiammine platinum(II) (CDDP) (Han et al. 2022), an effective broad-spectrum chemotherapeutic agent, has been approved by the U.S. Food and Drug Administration for solid tumor treatment for more than 40 years. Despite the significant role of CDDP-based drugs in cancer therapy, their toxicity is notable. While ototoxicity(Hu et al. 2020) and nephrotoxicity(Song et al. 2022; Pabla and Dong 2008) are well-documented due to their detectable nature, ovarian toxicity presents later and more insidiously, often progressing to irreversible ovarian reserve function loss before treatment begins. However, the precise mechanism of CDDP-induced ovarian toxicity remains largely unknown.

Premature ovarian failure (POF) (Fu et al. 2021), is an internal disease characterized by hypogonadism and decreased reserve function from menarche to 40 years old. POF affects 1%-5% of women of reproductive age, and it is the degree of ovarian damage that determines consequent diseases such as osteoporosis (Luo et al. 2018) and is strongly associated with infertility (Donnez 2020). Notably, the proportion of medical interventions (such as ovarian surgery, chemotherapy, and radiotherapy) contributing to the onset of POF is on the increase annually. Additionally, due to the lack of genetic models for assessing drug effectiveness, "CDDP-induced" chemotherapeutic drug models have become prevalent in researching POF mechanisms. Dysfunction of ovarian granulosa cells (GCs) is pivotal in POF development (Zhang et al. 2020a). The vulnerability of ovarian cells to damage may primarily stem from the similarities between the characteristics of ovarian functions and tumor cell survival, both being primary targets of chemotherapy. Ovarian primitive follicular cells cannot regenerate, leading to ovarian dysfunction and premature menopause when damaged. Currently, gonadotropin-releasing hormone agonists (Zong et al. 2022) are the primary treatment in the clinical setting for managing POF symptoms. Meanwhile, methods such as oocyte or ovarian tissue cryopreservation and embryo freezing are also employed as last-resort strategies to preserve female fertility (Kristensen et al. 2021). However, oxidative stress that is increased in the media by freezing and thawing could have an impact on the viability of the embryo. Freezing induces the hypothermia-hypoxia phenomena, which is linked to the decoupling of the internal mitochondrial membrane and is characterized by a decrease in ATP levels. This leads to an increase in superoxide free radical levels, which encourage the levels of reactive oxygen species (ROS) and cell apoptosis (Sayed et al. 2021). These interventions are merely temporary solutions and come with their own set of challenges, including complications, variable effectiveness, and high costs, which significantly restrict their clinical application (Dolmans et al. 2020). Thus, there is an urgent need to develop new treatments with minimal side effects for women to address ovarian damage.

Ferroptosis is thought to be a type of scheduled cell death that is driven by iron toxicity. It differs from other types of cell death both morphologically and biochemically. It is characterized by redox-active iron accumulation, glutathione depletion, and lipid peroxidation. It is controlled by particular metabolic pathways, such as cellular oxidative redox state, iron metabolism, and energy metabolism (Nassar et al. 2023). Recent studies indicate that ferrostatin-1 (FER-1), a ferroptosis inhibitor, can effectively mitigate CDDP-induced kidney injury (Liu et al. 2020). In vivo studies suggest that inhibiting Fe^2+^ accumulation can help alleviate ovarian damage in POF mice (Geng et al. 2022). Conversely, ((1S,3R)-RSL3) RSL3 promotes ferroptosis, leading to inflammation and cell death (Cui et al. 2021). The implications of ferroptosis and its regulators on CDDP-induced toxicity remain underexplored.

The present study aimed to analyze the role of ferroptosis in CDDP-induced ovarian injury, both in vitro and in vivo. Ferroptosis can play a key role in significantly contributing to CDDP-induced ovarian injury, and FER-1 may effectively counteract this injury. Through a series of in vitro and partial in vivo experiments, this study aimed to elucidate the regulatory mechanism of ferroptosis in CDDP-induced ovarian injury, offering a novel protective strategy for patients receiving chemotherapy.

## Material and methods

### Experimental animals and medication

Laboratory Animal Center of Harbin Medical University in Heilongjiang Province approved the protocols for animal experiments (No: YJSDW2023-107), and all methods were performed in accordance with ARRIVE guidelines and relevant and regulations.

Ten female Sprague Dawley (SD) rats (3 weeks old) were used in cellular experiments, and female SD rats (6 weeks old) with a normal estrous cycle were used for animal modelling, all animals sourced from the Department of Animal Science at Harbin Medical University. The cellular experiments rats were intraperitoneally injected with pregnant horse serum (80 IU/rat) for 24 h and then sacrificed by cervical dislocation. The bilateral ovaries were quickly removed, after that piercing follicle with a 1 ml syringe needle. Collect GCs, and cells were maintained in DMEM/F12 (GENEVIEW, China) supplemented with 10% FBS (Biological Industries, USA), at 37℃ with 5% CO_2_ atmosphere (Dong et al. 2023). The cell experiments were organized into six groups: control group (CON), FER-1 group, CDDP group, RSL3 group, RSL3 + CDDP group, and FER-1 + CDDP group. In addition, the FER-1 + RSL3 group was increased when cytotoxic concentrations were detected.

The experimental animals were categorized into three groups (n = 5 per subgroup): CON group, CDDP group, and CDDP + FER-1 group. Initially, the rats were randomly assigned to either the control group (n = 10) or the CDDP group (n = 20). In the control group, five rats were randomly selected to receive a saline intraperitoneal injection for 7 days, followed by an intraperitoneal injection of saline (2% dimethyl sulfoxide (DMSO), i.p.) three times a week for 2 weeks. The POF condition in rats was induced through an intraperitoneal injection of 2 mg/kg/day CDDP for 7 days. After 7 days, 10 rats with successfully induced POF were divided into two subgroups (n = 5 per subgroup): CDDP and CDDP + FER-1 (1 mg/kg, 2% DMSO), receiving injections of either saline (2% DMSO, i.p.) or FER-1 (1 mg/kg, 2% DMSO) three times a week for a total of 2 weeks, following the protocol described by Fang et al. (2019). After the treatment was finished, the experimental rats were anesthetized by i.p. injection of pentobarbital (50 mg/kg). Take blood from the abdominal aorta and collect them in centrifuge tubes. The blood was centrifuged (3000 r/min) for 15 min at RT to obtain the serum, and the ovaries were measured their weights and then stored at − 80℃ for subsequent experiments. All animals were sacrificed with pentobarbital (100 mg/kg) after surgery (Xue et al. 2022). The specific flow chart for animal modeling is shown in Fig. 4A, and the specific dosage formulated is shown in Supplementary Material S1.

### CCK8 assay

GCs from rat ovaries were isolated, seeded in 96-well plates, and cultured at 37 °C in a 5% CO_2_ atmosphere. Drug treatments included CDDP (10—40 μmol/L) for 24 h, RSL3 (1, 2, 3, and 5 μmol/L), FER-1 (0.5, 1, 2, 5, 10, 20, 30, and 40 μmol/L) for 2 h. The CDDP concentration was chosen based on previous research by our group (Dong et al. 2023). For the CCK8 assay, 5 μL of CCK8 solution (ApexBio, USA) was added to each 100 μL of the medium, incubated for 2 h, and the absorbance (Abs) was measured at 450 nm. Cell viability was expressed as a percentage of the control.

### Fe^2+^ detection

Intracellular and mitochondrial iron levels were determined using FerroOrange and Mito-FerroGreen (Dojindo, Japan), respectively. After treatment, GCs were stained with 1 μmol/L FerroOrange or 5 μmol/L Mito-FerroGreen at 37 ℃ for 30 min in the dark. Images were acquired using a confocal laser scanning microscope (Leica TCS-SP5II, Germany), and the experiment was repeated three times. Fluorescence intensity was analyzed using ImageJ software, with the average fluorescence intensity of each group being normalized to that of the control group.

### ROS detection

Intracellular and mitochondrial ROS levels were assessed using DCFH-DA (ApexBio, USA) and MitoSox-Red (MKBIO, China), respectively. GCs were treated under specified conditions in confocal Petri dishes, then double-stained with 50 μmol/L DCFH-DA and 5 μmol/L MitoSox-Red for 30 min in the dark. Afterward, cells were stained with Hoechst33342 (Solarbio, USA) for 10 min. Images were captured using a confocal laser scanning microscope (Leica TCS-SP5II, Germany) equipped with an oil immersion objective. Fluorescence intensity was measured with ImageJ and normalized to the average intensity of the control group.

### MMP measurement

To visualize mitochondrial membrane potential (MMP), GCs were cultured in confocal petri dishes and exposed to specific conditions. Following these treatments, the cells were stained with 10 μM Rhodamine123 (Solarbio, USA) in accordance with the manufacturer’s instructions. After incubation for 30 min at 37℃ in the dark, the GCs were further stained with Hoechst33342 for 10 min. They were then washed with phosphate-buffered saline and imaged using a confocal laser scanning microscope (Leica TCS-SP5II, Germany) equipped with an oil immersion objective. Images were chosen from random fields of view across three independent experiments.

### TEM

Cells were fixed in 2.5% glutaraldehyde in 0.1 M phosphate buffer (pH 7.4) for 2 h at 4℃, followed by post-fixation in 1% osmium tetroxide for 1 h. The samples were dehydrated through a graded ethanol series and embedded in Spurr’s resin. Ultrathin Sects. (60–80 nm) were cut using an ultramicrotome, mounted on copper grids, and stained with uranyl acetate and lead citrate. Transmission electron microscopy (TEM) (Hitachi HT7700, Japan) at 80 kV and images were captured with a CCD camera using for observe the cellular structures and mitochondrial.

### Estrous cycle measurement

Rats underwent daily swabbing and were treated with paraformaldehyde and crystal violet solution before microscopic examination for estrous cycle changes.

### ELISA

The concentrations of estradiol (E_2_) and follicle-stimulating hormone (FSH) were measured using enzyme-linked immunosorbent assay (Shanghai Enzyme-linked Biotechnology, China) following the manufacturer's instructions. The absorbance (Abs) for each group was recorded at 450 nm, and the serum levels of E_2_ and FSH were determined using a standard curve.

### Organ index

Rats were monitored, and their body weights were recorded at four intervals: before treatment, after model establishment, after 1 week of treatment, and at the end of the treatment period. The indices for both ovaries and uteri were then calculated.

### Tissue *iron* assay

Tissue iron levels were assessed using the iron assay kit (Leagene, China). Samples were processed, and their absorbance was measured at 562 nm with an enzyme meter (BIOTEK SYNERGY-H1, USA). The results were then calculated.

### MDA/SOD/GSH assay

The levels of malondialdehyde (MDA), superoxide dismutase (SOD), and glutathione (GSH) were determined using respective assay kits (Beyotime Biotechnology, China), SOD assay kit (Nanjingjiancheng Bioengineering, China) and GSH assay kit (Beyotime Biotechnology, China). After sample processing, absorbance was measured, and the results were calculated according to kit instructions using an enzyme meter (BIOTEK SYNERGY-H1, USA).

### Western blot

Lysate preparation and western blot analysis were conducted following established protocols (Zhang et al. 2023a). Ovarian tissues were lysed in radioimmunoprecipitation assay lysis buffer to extract total proteins, which were then transferred onto polyvinylidene difluoride membranes. Initially, membranes were blocked with 1% bovine serum albumin for 90 min, washed thrice with Tris-buffered saline with Tween 20 (TBST), and incubated with primary antibodies overnight at 4℃. The primary antibodies used were as follows: KEAP-1 (1:1500, #wl03285, Wanlei Biotechnology, China), NRF2 (1:1000, #bs1258, Bioworld Technology, USA), HO-1 (1:1000, #WL02400, Wanlei Biotechnology, China), GPX4 (1:1000, #ab125066, Abcam, UK) and GAPDH (1:1000, #WH199424, ABclonal Technology, China). Following TBST washes, the membranes were incubated with secondary antibody HRP(H + L) IgG (#WH184227, ABclonal Technology, China) for 90 min at room temperature. Detection was performed using an automatic electrochemiluminescence analysis system (BIO-RAD AI-600, USA) was used to show the blot bands.

### Molecular docking simulation

The three-dimensional structure of NRF2 for molecular docking was obtained from AlphaFold database (https://alphafold.ebi.ac.uk) (UniProt: a hub for protein information 2015) and the structure of GPX4 was obtained from PubChem (http://pubchem.ncbi.nlm.nih.gov/) (Hähnke et al. 2018) and PyMOL2.6.0 (PDB: 2OBI). FER-1 was docked into GPX4 or NRF2 using Molecular Operating Environment 2019 software (Liu et al. 2017) with number of operations selected by 50 times. Thus, molecular docking scores was assessed based on the magnitude of binding energy and the results were visualized by PyMOL and Discovery studio 2019 software.

### Data analyses

Statistical analysis of experimental data was conducted using GraphPad Prism 9.4.1 and IBM SPSS Statistics 25 software. Data were presented as mean ± standard deviation. Statistically significant differences between the mean of multiple groups of independent samples were analyzed using one-way analysis of variance, and the LSD method was used for pairwise comparison between groups. P-value < 0.05 was considered statistically significant.

## Results

### Inhibition of ferroptosis ameliorates CDDP-induced GCs injury

**Fig. 1:**
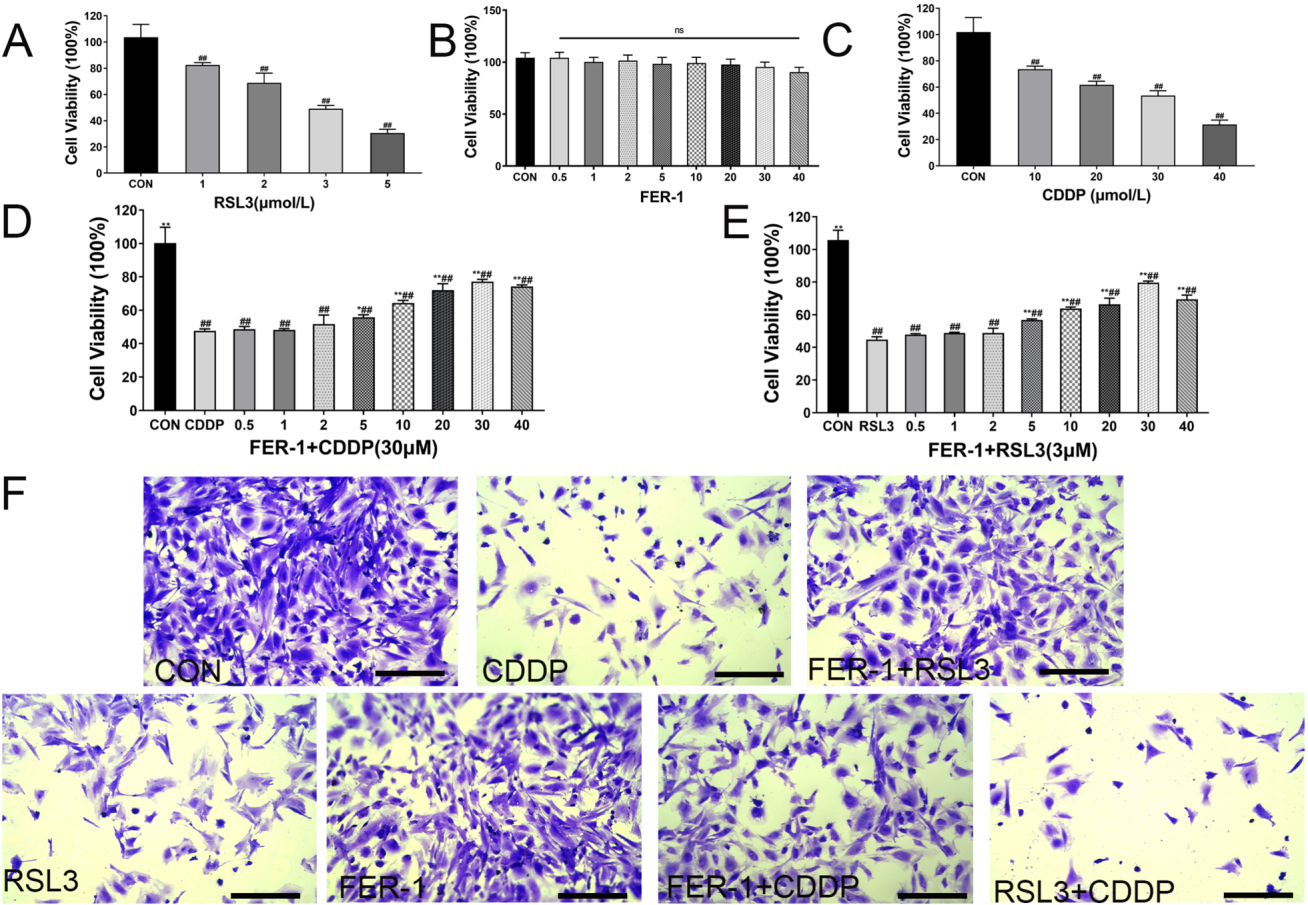
CDDP and RSL3 induced ferroptosis in GCs. Cells treated with varying concentrations of RSL3 (**A**), FER-1 **B** for 2 h or CDDP **C** for 24 h were analyzed by CCK‐8 assays. Cells were pre‐treated with varying concentrations of FER‐1 for 2 h, followed by addition of 30 μmol/L CDDP for 24 h **D** or 3 μmol/L RSL3 for 2 h **E** and analyzed by CCK-8 assays. **F** Cell ability of different groups by crystalline violet assay (purple color). Scale bar: 200 μm. All the data represent the mean ± SD. of three independent experiments. ^#^P < 0.05, ^##^P < 0.01 vs the CON group; *P < 0.05, **P < 0.01 vs the group treated with RSL3 (C) or CDDP (D) alone

The data indicated that RSL3 decreased cell viability in a dose-dependent manner (Fig. 1A), while different concentrations of FER-1 did not affect cell viability (Fig. 1B). Notably, 3 μmol/L of RSL3 was identified as the optimal concentration for future experiments. CDDP significantly reduced GC viability in a dose-dependent manner, causing severe cytotoxicity (Fig. 1C). A concentration of 30 μmol/L was determined for inducing GC injury in subsequent experiments.

GCs were pretreated with FER-1 for 2 h before being exposed to 30 μmol/L CDDP for 24 h or 3 µmol/L RSL3 for 2 h (Fig. 1D, E). The 30 μmol/L concentration of FER-1 offered the most significant protection against damage from CDDP or RSL3, making it the chosen concentration for further studies. The crystalline violet assay corroborated that CDDP and RSL3 were cytotoxic to GCs, and pre-treatment with FER-1 could reverse this cell damage (Fig. 1F).

## Inhibition of ferroptosis improves CDDP-induced *iron* overload in GCs

**Fig. 2:**
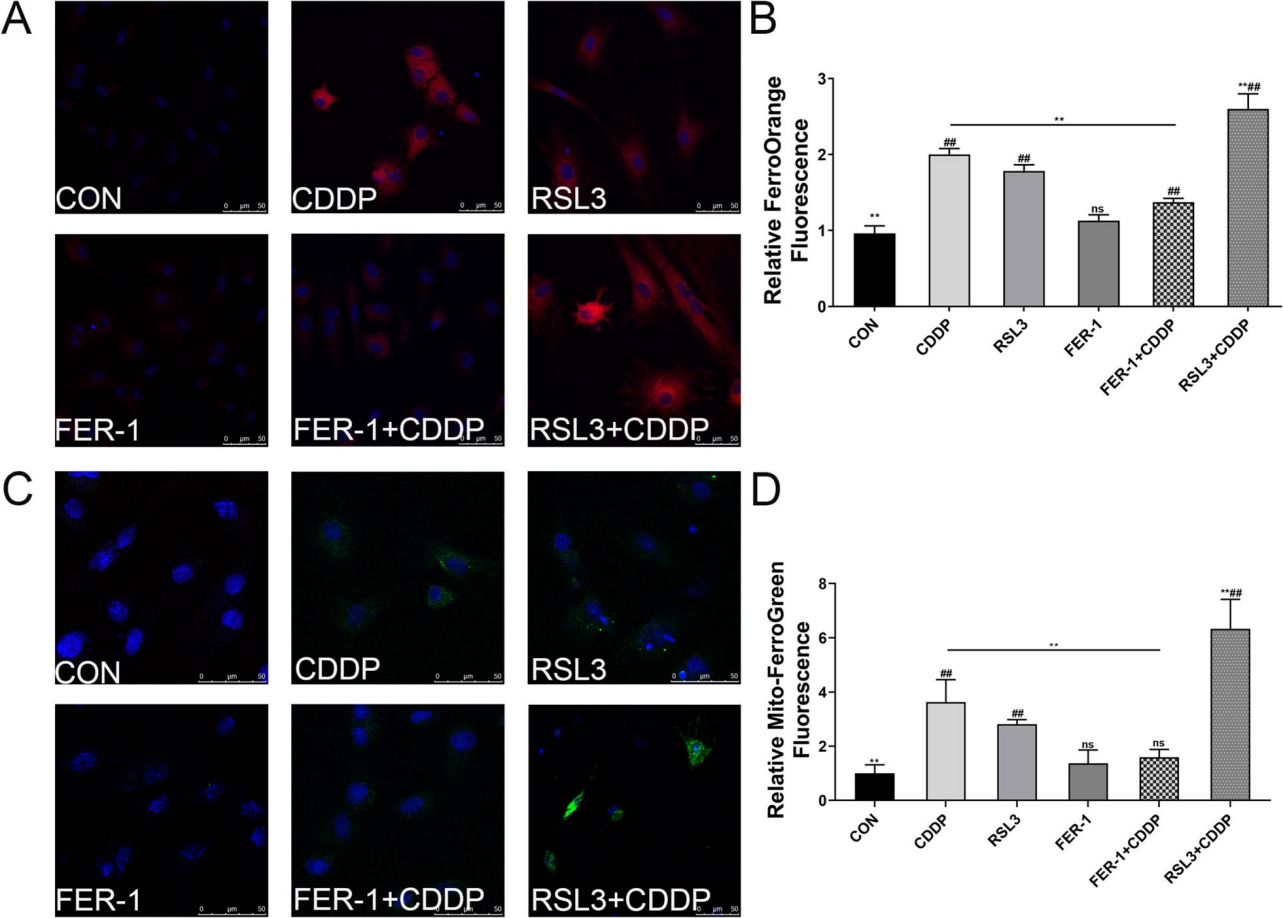
Effect of FER‐1 on iron production in CDDP‐damaged GCs. **A** Intracellular Fe^2+^ detected by FerroOrange (red color). **B** Relative fluorescence intensity of FerroOrange. **C** Mitochondrial Fe^2+^ assayed by Mito-FerroGreen (green color). **D** Relative fluorescence intensity of Mito-FerroGreen. Scale bar: 50 μm. The fluorescence intensity was quantified by ImageJ software. The data are shown as mean ± SD. *P < 0.05, **P < 0.01 and ns (no significant) vs the CDDP group; # P < 0.05 and ^##^P < 0.01 vs the CON group

After co-localization of the FerroOrange and Mito-FerroGreen probes, respectively, with Hoechst 33342, the result illustrates that CDDP treatment prompted ferroptosis in GCs, as evidenced by elevated FerroOrange (Fig. 2A, B) and Mito-Ferrogreen (Fig. 2C, D) signals compared with the control group. FER-1 treatment significantly mitigated these effects, which were further exacerbated by pretreatment with RSL3.

### Fer-1 prevented oxidative stress and loss of MMP concentration in GCs induced by CDDP

**Fig. 3:**
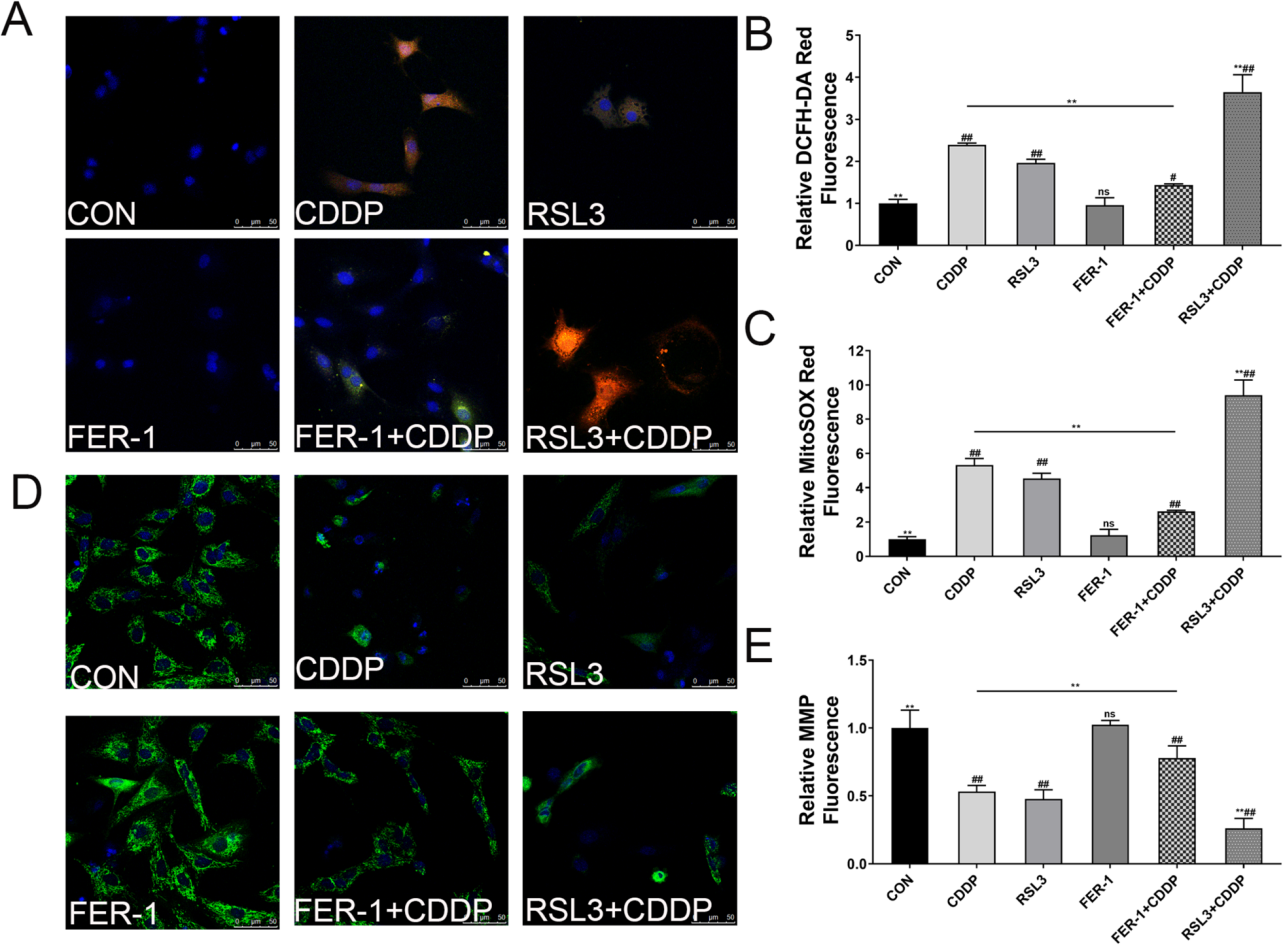
Effect of FER-1 on ROS generation and MMP in CDDP‐damaged GCs. Representative images of GCs stained by **A** DCFH-DA (green color), **A** MitoSox-Red (red color) and **D** Rhodamine123 (green color). Scale bar:50 μm. Relative fluorescence intensity of DCFH‐DA (**B**), MitoSox-Red **C** and Rhodamine123 **E** fluorescent probes. The data are shown as mean ± SD of three independent experiments. **P < 0.01 and ns vs the CDDP group; ^##^P < 0.01 vs the CON group

Staining results indicate that ROS levels were significantly elevated in cells pretreated with RSL3 compared with those treated with CDDP only. Conversely, pretreatment of cells with FER-1 significantly reduced CDDP-mediated ROS overproduction (Fig. 3A, B). Mitochondrial ROS levels paralleled changes in intracellular ROS levels, with FER-1 pretreatment diminishing CDDP-induced mitochondrial ROS levels (Fig. 3A, C).

The fluorescence intensity in the CDDP group was markedly reduced compared with the control group, indicating a loss of membrane potential. This loss was intensified with RSL3 treatment. However, FER-1 pretreatment protected the cells by reducing mitochondrial damage and improving fluorescence intensity after CDDP exposure (Fig. 3D, E).

### FER-1 inhibited ferroptosis-associated morphological alterations in GC cells induced by CDDP

**Fig. 4:**
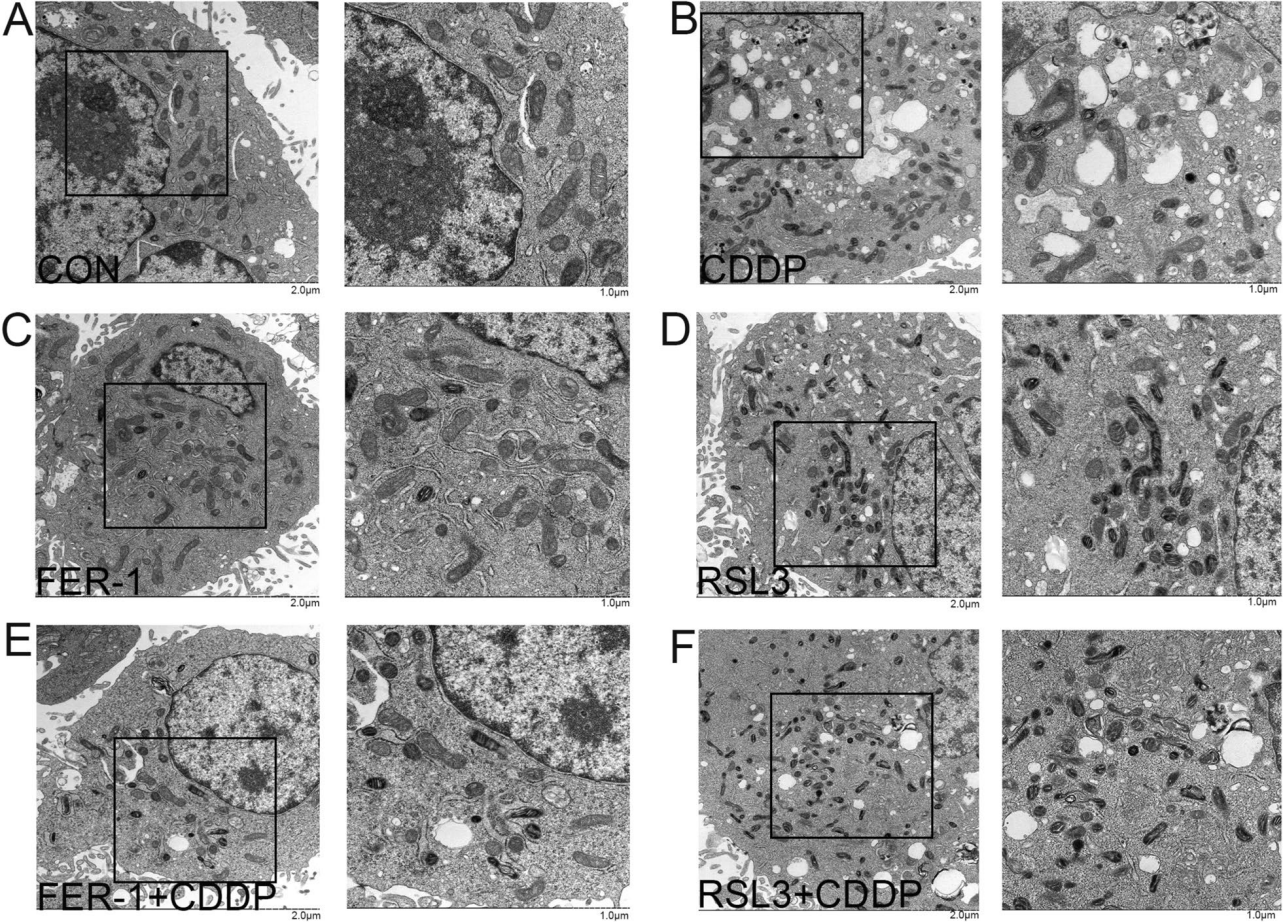
Effect of FER-1 on CDDP‐induced GCs by TEM. **A**–**F** represent the cellular structure and mitochondrial morphology under electron microscopy for different groups. Scale bar: Each group consists of two images, from left to right, measuring 2 μm and 1 μm respectively

The separate application of CDDP (Fig. 4B) or RSL3 (Fig. 4D) results in the loss of normal mitochondrial morphology in GC cells, accompanied by characteristic morphological changes indicative of ferroptosis, such as increased electron density of the mitochondrial double membrane, reduced mitochondrial cristae, and rupture of the mitochondrial membrane. In contrast, the combined treatment with CDDP and RSL3 exacerbates the ferroptosis state, leading to increased mitochondrial density and reduced mitochondrial volume. Conversely, when FER-1 is administered to prevent the effects of CDDP, a significant portion of the mitochondrial morphology is restored to a normal state, with a marked improvement in mitochondrial distortion.

### FER-1 alleviated CDDP-induced ovary injury in vivo

**Fig. 5:**
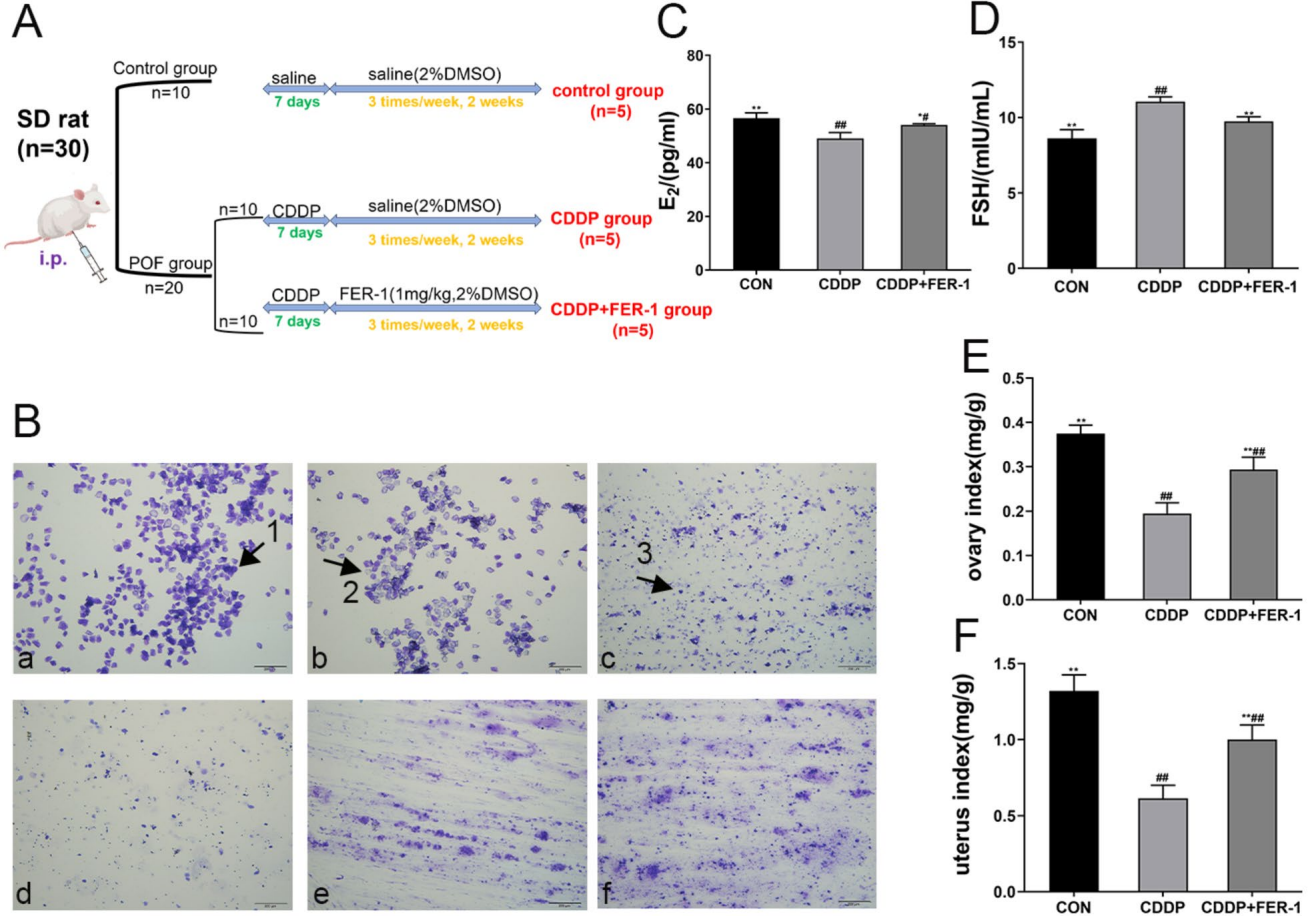
Effect of FER-1 on CDDP induced ovary injury in vivo. **A** is the specific flow chart of experimental modeling. **B** is the rat estrous cycle (purple color) and scale bar is 200 μm; (a) is the pre-estrus period; (b) is the estrus period; (c) is the post-estrus period; (d) is the inter-estrus period; (e) is the simulated disturbed estrous cycle; (f) is the estrous cycle two weeks after FER-1 treatment. Arrow 1 points to nucleated epithelial cells, arrow 2 to non-nucleated epithelial cells, and arrow 3 to leukocytes. **C**, **D** are the level of serum E_2_ and FSH hormones. **E** is the level of ovarian index in different groups of rats. **F** is the level of uterine index in different groups of rats. The data are shown as mean ± SD. of three independent experiments. *P < 0.05, **P < 0.01 and ns vs the CDDP group; ^#^*P* < 0.05, ^##^*P* < 0.01 vs the CON group

The findings revealed significant estrous cycle disruptions in the CDDP model compared with normal rats, which were nearly normalized after FER-1 treatment (Fig. 5B). Serum hormone levels of E_2_ were significantly reduced, and FSH was significantly increased in the CDDP group compared to the control group. FER-1 treatment improved these hormone levels (Fig. 5C, D). Furthermore, CDDP caused weight loss and decreased ovarian and uterine indices in rats; conversely, FER-1 treatment ameliorated these conditions (Fig. 5E, F).

### Fer-1 alleviates ferroptosis from CDDP-induced oxidative stress in vivo

**Fig. 6:**
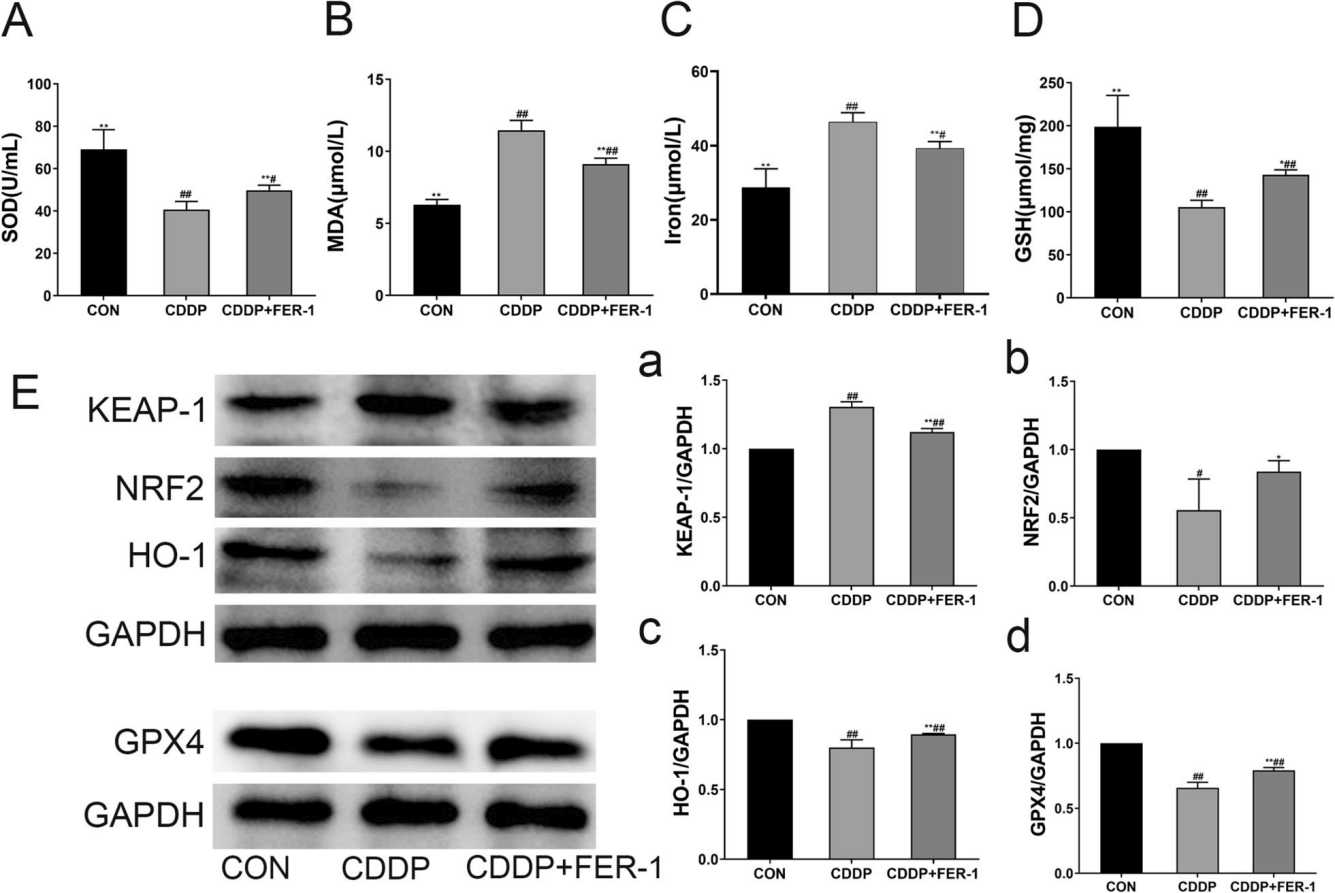
FER-1 protects CDDP-induced ferroptosis in rat POF model. After CDDP-induced rat POF model treatment with FER-1, ovarian tissue of **A** SOD, **B** MDA, **C** Iron and **D** GSH content. **E** Ovary proteins were extracted and subjected to Western blot analysis for (**a**) KEAP-1, (**b**) NRF2, (**c**) HO-1 and (**d**) GPX4. The data are shown as mean ± SD. of three independent experiments. *P < 0.05, **P < 0.01 and ns vs the CDDP group; ^#^P < 0.05, ^##^P < 0.01 vs the CON group

CDDP significantly increased iron and MDA content in ovarian tissues while reducing SOD and GSH levels. FER-1 treatment decreased iron and MDA levels (Fig. 6A–D). To determine the role of iron metabolism in the ovarian toxicity of CDDP, several iron metabolism markers were evaluated. Compared with the control group, the KEAP-1 expression level was elevated in the CDDP group, and the protein expression of NRF2, HO-1, and GPX4 was significantly reduced; FER-1 treatment lowered KEAP-1 expression and increased the expression of NRF2, HO-1, and GPX4 (Fig. 6E).

### FER-1 binds to GPX4 or NRF2

**Fig. 7:**
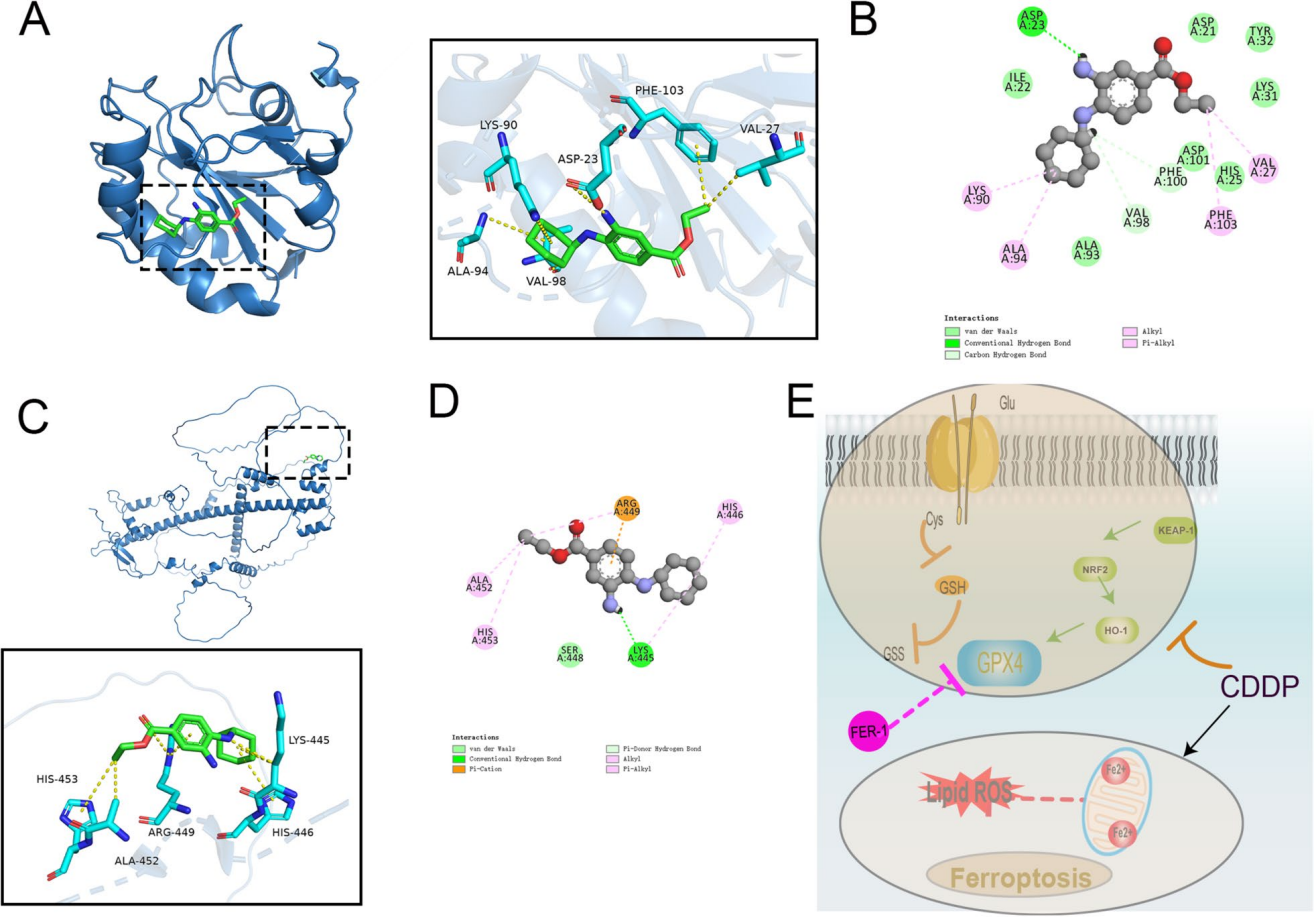
FER-1 binds to GPX4 or NRF2. **A** 3D and **B** 2D diagram of the interaction between FER-1 and GPX4. **C** 3D and **D** 2D diagram of the interaction between FER-1 and NRF2. **E** Schematic illustration of the proposed GPX4-related mechanisms

FER-1 strongly bound to residues GPX4’s Gly284 and Val125 by hydrogen bonds (Fig. 7A, B) with a high binding energy (− 5.7126 kcal/mol). FER-1 strongly bound to NRF2’s residues Lys445 by hydrogen bonds (Fig. 7C, D) with a low binding energy (− 4.6260 kcal/mol).

## Discussion

This study employed GCs and a POF rat model to demonstrate that ferroptosis, an iron-dependent form of oxidative cell death, significantly contributes to the development of CDDP-induced ovarian damage. Additionally, FER-1 mitigated CDDP-induced ovarian toxicity by inhibiting ferroptosis and oxidative stress. These findings suggest that targeting ferroptosis may offer a novel therapeutic strategy for treating CDDP-induced ovarian toxicity.

GCs, the largest and primary functional cell population within the follicle, engage in bidirectional signaling with developing oocytes in the ovarian microenvironment. This interaction supports oocyte development and promotes follicle formation (Chen et al. 2022). Throughout the physiological process of follicle formation, the activation and sustained growth of oocytes rely on the nutritional and paracrine functions provided by surrounding GCs (Zhang et al. 2022). Abnormalities in GCs lead to oxidative stress and mitochondrial disorders, potentially causing POF in females. The most direct evidence of CDDP's ovarian toxicity is its detrimental effect on ovarian GCs when used to treat patients with cancer (Cui et al. 2020). Numerous studies indicate that CDDP interacts covalently with DNA's purine bases, creating cross-links that inhibit protein translation and synthesis, leading to apoptosis (Chen et al. 2021; Lin et al. 2023). In addition, the primary target of excessive oxidative loading caused by CDDP is the mitochondria, and mitochondrial dysfunction due to oxidative loading causes cell death and generates ROS derived from mitochondrial metabolism (Nagasawa et al. 2021). Recent research has linked ferroptosis with CDDP-induced cell death (Gao et al. 2022; Zhang et al. 2023b; Du et al. 2023); however, the role of small-molecule inhibitors of ferroptosis in mitigating CDDP-induced toxicity had not been previously reported. The study showed that ferroptosis is a key mechanism underlying CDDP-induced ovarian damage. FER-1, a specific inhibitor of ferroptosis, offers antioxidant benefits and shields cells from CDDP damage. In this study, we have chosen FER-1 as the therapeutic drug. Previous studies have shown that FER-1 also exhibits favorable efficacy against cisplatin-induced ototoxicity (Hu et al. 2020). Similar as FER-1, liproxstatin-1 acts as an RTA with higher potency in phospholipid bilayers than in organic solvent compared to phenolic RTAs. On the one side, the antioxidant capacity of Fer-1 was lower than the well-established lipophilic antioxidant vitamin E. the RTA activity of Fer-1 was significantly better than vitamin E. In addition, Fer-1 can stabilize radicals since the newly formed radical can trap additional ROS (Zilka et al. 2017). Conversely, RSL3, a ferroptosis inducer, exacerbates CDDP-induced cellular damage by promoting iron death in GCs.

Our findings indicated that both RSL3 and CDDP reduce the viability of GCs in a dose-dependent manner. Notably, CDDP's detrimental effect was greater than that of RSL3, possibly due to additional modes of cell death involved in the damage induced by CDDP. Conversely, FER-1 mitigated this cell damage and decreased the severity of the damage. These observations suggest that GCs are prone to ferroptosis in vitro, and activating this novel form of cell death compromises cell viability. This aligns with the hypothesis that ferroptosis contributes to the damage CDDP inflicts on GCs. Furthermore, blocking ferroptosis with FER-1 reduced the harm to GCs, corroborating the findings of prior research (Shi et al. 2022).

Highly reactive iron ions are a major cause of cell damage and death (Masaldan et al. 2018). Intracellular free iron predominantly exists as Fe^2+^ and Fe^3+^, with Fe^2+^ playing a crucial role in the intracellular reducing environment, metal transport, and water solubility (Venkataramani 2021). Iron is essential for mitochondrial function as part of the iron-sulfur cluster in the electron transport chain. Mitochondrial dysfunction can lead to excess iron ions, and the two factors are interdependent (Battaglia et al. 2020). Our findings suggest that CDDP induces ferroptosis in GCs by disrupting iron homeostasis, leading to significant Fe^2+^ accumulation. While RSL3 exacerbates this imbalance, FER-1 prevents the onset of iron disorders. Given that ferroptosis is largely dependent on intracellular iron accumulation, this study initially measured levels of intracellular Fe^2+ ^and mitochondrial Fe^2+ ^using. We suggested that Fe^2+^ may enters GCs through endocytosis, mediated by peripheral iron carrier proteins. Iron overload, resulting from insufficient iron carrier proteins, triggers GC dysfunction (Li et al. 2020). However, iron metabolism disorder reduces intracellular iron storage, and Fe^2+^ facilitates hydroxyl radical production, promoting ferroptosis (Stockwell 2022).

ROS play a critical role in maintaining the normal fluctuations within biological systems. Specifically, the production of ROS within cells and mitochondria triggers certain cellular responses, such as the removal of dysfunctional mitochondria and cells, to protect adjacent mitochondria and cells from damage (Zorov et al. 2014). However, uncontrolled ROS levels can lead to severe cellular damage, unnecessary cell death, and ultimately, failure of entire organs. As ROS accumulation is closely linked to ferroptosis, total ROS and intra-mitochondrial ROS production were evaluated, respectively. In addition, mitochondrial membrane potential and morphological changes of mitochondria were observed. Our studies indicate that CDDP elevates ROS production in GCs cells and mitochondria, while RSL3 further increases ROS production. Both compounds cause mitochondrial damage, including decreased MMP and changes in mitochondrial morphology. Conversely, FER-1 not only significantly reduced the excessive production of ROS in cells and mitochondria, but also alleviated mitochondrial damage. Our result shows that excessive oxidative stress in GCs results in increased ROS levels and mitochondrial dysfunction, which are key contributors to ovarian damage (Ni et al. 2022). This aligns with our findings from measuring iron (Fe^2+^) levels, suggesting that activating ferroptosis in GCs—by disrupting mitochondrial dynamics—can promote inflammation and worsen oxidative stress. Inhibition of ferroptosis can mitigate mitochondrial dysfunction and restore normal cellular physiological activities.

Further investigations explored the effect of ferroptosis on ovarian damage induced by CDDP through in vivo studies. The ovarian endocrine function, governed by the gonadal axis through positive and negative feedback, can become disrupted, leading to perimenopausal disorders and premature menopause (Zhang et al. 2020b). Ferroptosis accelerates cellular exhaustion and tissue aging, affecting tissue development (Zheng et al. 2021). To examine whether FER-1 pretreatment also shields ovarian tissues from CDDP-induced injury through effects on the rat estrous cycle, serum hormone levels, and organ indices, a POF rat model was established. Our observations show that CDDP treatment disrupts endocrine function and increases oxidative stress in rats, causing tissue damage. These findings are consistent with cells part’s results, highlighting the detrimental effects of CDDP on rat gonads. We suggested that the presence of ferroptosis in CDDP-induced endocrine damage in rat ovaries leads to decreased inhibin levels, failing to suppress FSH, which, in turn, negatively affects E_2_ secretion. This results in high FSH and low E_2_ levels. FER-1 treatment significantly counteracts these effects, indicating that inhibiting ferroptosis could aid in recovering ovarian endocrine function.

Lipid peroxidation, driven by ROS, can damage cell membrane composition and structure, producing large amounts of lethal MDA and SOD (Zhang et al. 2020c). The GSH-Fe^2+^ complex plays a crucial role in maintaining iron homeostasis within the cell (Zheng and Conrad 2020). Our findings demonstrate that CDDP significantly increases MDA and tissue iron levels while reducing SOD and GSH levels. However, FER-1 treatment reverses these changes, suggesting CDDP acts as a ferroptosis agonist, inhibiting antioxidant activities and enhancing iron content. We believe that iron ions catalyze lipid peroxidation of the cytosol membrane through the "Fenton" reaction, generating excessive ROS and causing oxidative damage in the ovary. These results underscore the importance of understanding ferroptosis mechanisms in treating ovarian damage.

Normally, NRF2's activity is moderated by KEAP-1. Under stress, NRF2 activates genes such as HO-1 and GPX4 by binding to antioxidant response elements in their promoter regions, which helps mitigate oxidative stress. HO-1 transforms free hemoglobin into Fe^2+^. Reduced NRF2 levels diminish HO-1 activity, causing excessive hemoglobin breakdown and Fe^2+^ accumulation, thereby triggering ferroptosis (Luo et al. 2022).This diminishes HO-1 and GPX4 expression, exacerbates oxidative damage in ovarian tissues, and promotes ferroptosis. By contrast, FER-1 increased the binding of KEAP-1 to NRF2, leading to NRF2 overexpression, which largely inhibited lipid peroxidation, ameliorated oxidative stress, and inhibited ferroptosis in the ovarian tissue. Previous studies have demonstrated that GPX4 and NRF2 are key genes involved in the pathological process of ferroptosis and are regarded ferroptosis-specific markers. The molecular docking results demonstrate that GXP4 exhibits a significantly better binding affinity with FER-1 as compared to nrf2.Therefore, GPX4-related pathways ware a possible potential target of FER-1, and targeting GPX4 might be a promising ovary protective strategy. GPX4 can catalyze GSH to GSS via the oxidative reaction, during which lipid peroxides are reduced into their less-reactive alcohol form. GPX4 could terminate the ferroptotic cascade by using GSH as the substrate (Deng et al. 2020).Recently, accumulating evidence revealed that a close link between Nrf2/HO-1 pathway and GPX4. GPX4, directly regulated by NRF2 and central to ferroptosis regulation, participates in forming the GSH/GPX4 axis, showcasing significant antioxidant properties in preventing ferroptosis (Chen et al. 2023). Our findings indicate that CDDP enhances KEAP-1 expression, leading to increased NRF2 degradation and reducing total GSH level. Therefore, we propose that FER-1 may inhibit the occurrence of CDDP-induced ovarian toxicity by affecting the progression of ferroptosis through two signaling pathways: GSH/GPX4 and NRF2/HO-1/GPX4.

Our research points to iron regulation as a potential therapeutic approach for countering CDDP-induced ovary injury. We chose rats as our experimental model. On one hand, rats have similar ovarian morphology to humans and exhibit a certain estrous cycle, making them the preferred animal model for POF. Therefore, we believe that the experimental data in this study will have certain implications for future clinical applications. It is worth noting that the estrous cycle in rats is much shorter than that in humans, and they can release multiple eggs simultaneously. It may be necessary to use other mammalian animals to further validate our data and ultimately achieve clinical translation. In this study, the dosage of fer-1 we used belongs to a relatively low dose. However, for clinical treatment, high-dose medication can be used for short-term impact therapy, which may yield better results. However, the potential adverse reactions of FER-1 still need to be explored. Furthermore, long-term use of FER-1 requires attention to its pharmacokinetic and pharmacodynamic characteristics, as the potential side effects of FER-1 remain unknown. If long-term use of this drug is needed, alternative methods such as the use of polymer materials may be considered in the future. Therefore, we plan to conduct animal experiments with long-term treatment in the future to further validate our findings. Although we have used molecular docking to predict the interaction between FER-1 and GPX4, in the future, we will use gene mice to validate our hypotheses regarding the underlying mechanisms. In addition, the methods primarily used in this study were semi-quantitative. It is necessary to further employ quantitative detection methods such as flow cytometry to determine the extent of ferroptosis.

## Conclusions

In summary, the study provided the first evidence that ferroptosis plays a critical role in the toxicity of CDDP in the ovary. FER-1 could inhibit CDDP-induced ovarian injury through the ROS-mediated ferroptosis pathway. Additionally, activating GPX4 to prevent ferroptosis represents a promising therapeutic target for treating CDDP-induced acute ovarian injury in clinical settings.

## Supplementary Information

Supplementary Material 1.

## Data Availability

All data presented in the present study are available from the corresponding author on reasonable request.

## Abbreviations

FER-1: Ferrostatin-1
CDDP: Cis-dichlorodiammineplatinum(II)
RSL3: (1S,3R)-RSL3)
POF: Premature ovarian failure
GCs: Granulosa cells
CCK-8: Cell counting kit-8
SD: Sprague Dawley
