# Supplementary material for: Ferrostatin-1 ameliorates Cis-dichlorodiammineplatinum(II)-induced ovarian toxicity by inhibiting ferroptosis
Source: Mol Med. 2024 Sep 13;30:150. doi: 10.1186/s10020-024-00923-7 (PMC11401273; doi:10.1186/s10020-024-00923-7)
Supplement: Supplementary file 1 — Supplementary Material 1. [file 10020_2024_923_MOESM1_ESM.docx]

**Supplementary material**

**Title**

Ferrostatin-1 ameliorates Cis-dichlorodiammineplatinum(II)-induced ovarian toxicity by inhibiting ferroptosis

**Author name**

Lu Zhang^12^, Zhe Dong^2^, Fan Jiang^2^, Huaju Huang^2^, Hui Ding^1^, Meimei Liu^12*^

**Author affiliation**

^1^Harbin Medical University, Harbin, 150086 Heilongjiang Province China

^2^Department of Obstetrics and Gynecology, The Second Affiliated Hospital of Harbin Medical University, Harbin, 150086 Heilongjiang Province China

^3^Jinzhou Medical University, Jinzhou, 121000 Liaoning Province China

**Author information**

Lu Zhang, Email: zhang932612@163.com.

Zhe Dong, Email: jzykdxdongzhe@163.com

Fan Jiang, Email: jf652958055@icloud.com

Huaju Huang, Email: huang18809886340@163.com

Hui Ding, Email: 202203027@hrbmu.edu.cn

Meimei Liu, Email: hydliumeimei@163.com

* Corresponding author.

**S1 The ratio of FER-1 in POF animal model**

Each of our rats weighed about 200 g on average, and the injection dosage was 1 mg/kg. At the same time, we dissolved 1 mg fer-1 in 100 μl DMSO to form a reserve solution of 10 mg/ml, and then added saline to form a working solution containing 2% dmso for intraperitoneal injection. The total volume of each injection per rat was 1 ml.

The method we use to administer i.p. injections to rats and FER-1’s concentration is borrowed from Dr. Fang, who published and patented the same method in 2017, The specification of the patent (A) and the original content of article (B) are listed below. (Figure R2)

Reference：

Fang X, Wang H, Han D, Xie E, Yang X, Wei J, Gu S, Gao F, Zhu N, Yin X, Cheng Q, Zhang P, Dai W, Chen J, Yang F, Yang HT, Linkermann A, Gu W, Min J, Wang F. Ferroptosis as a target for protection against cardiomyopathy. Proc Natl Acad Sci U S A. 2019 Feb 12;116(7):2672-2680. doi: 10.1073/pnas.1821022116. Epub 2019 Jan 28. PMID: 30692261; PMCID: PMC6377499.

| A  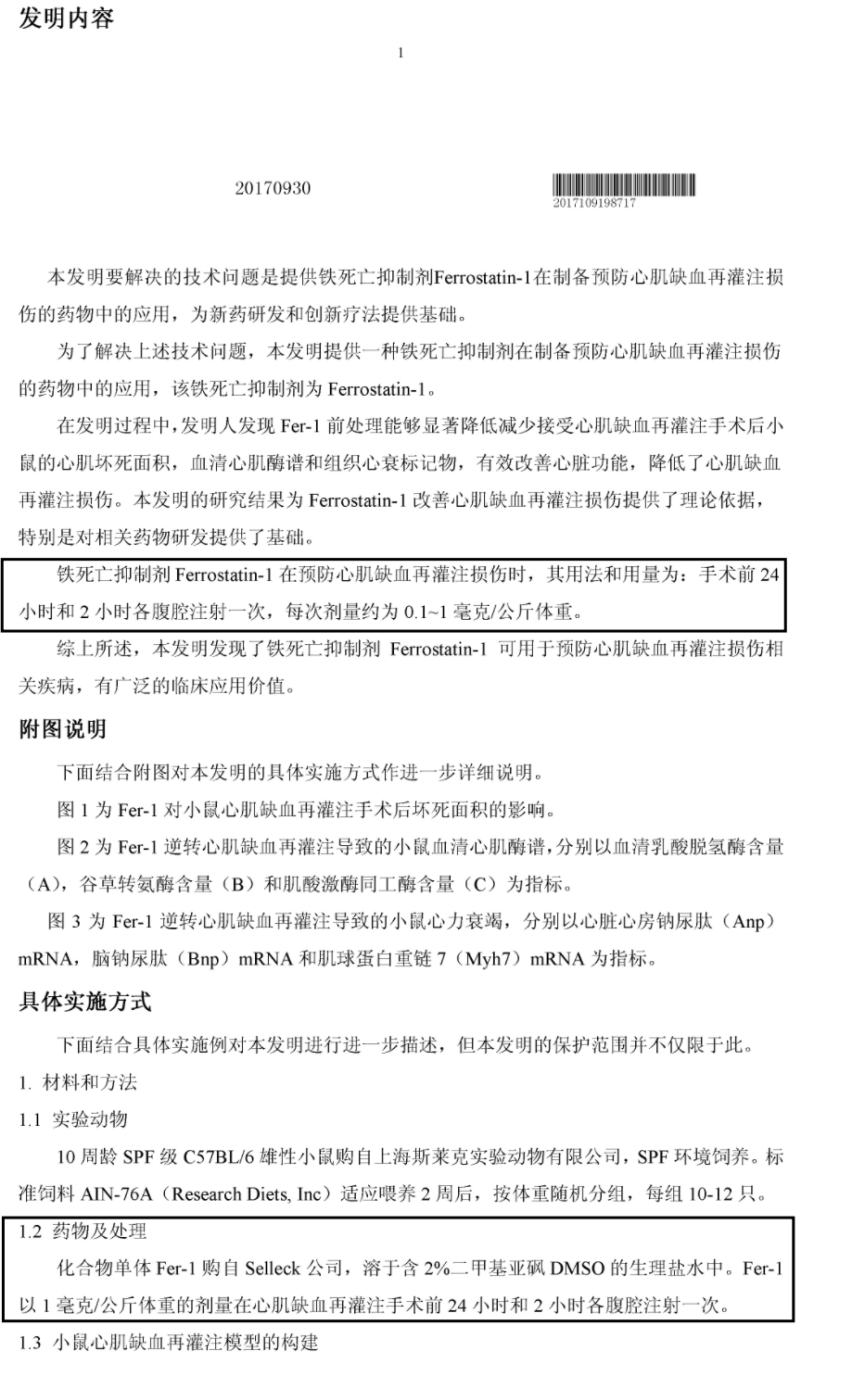 | **B**  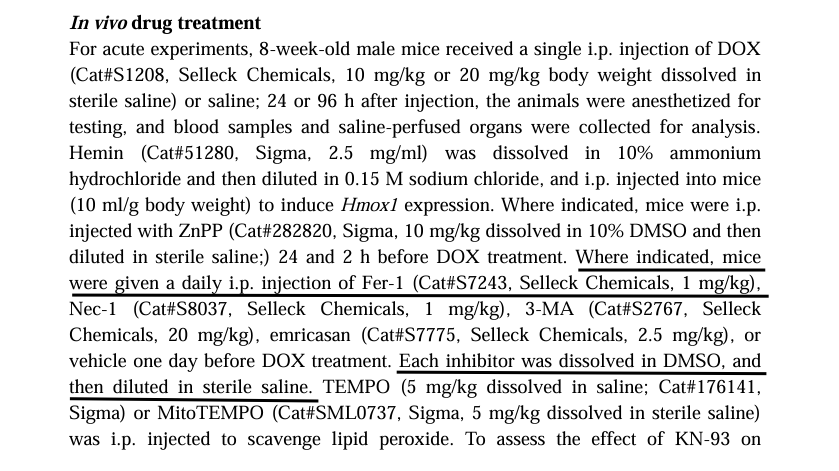 |
| --- | --- |
| Figure S1 Source of Fer-1 and DMSO matching doses. (A) The specification of the patent (B) The original content of article | |

**S2 Organ tissue**


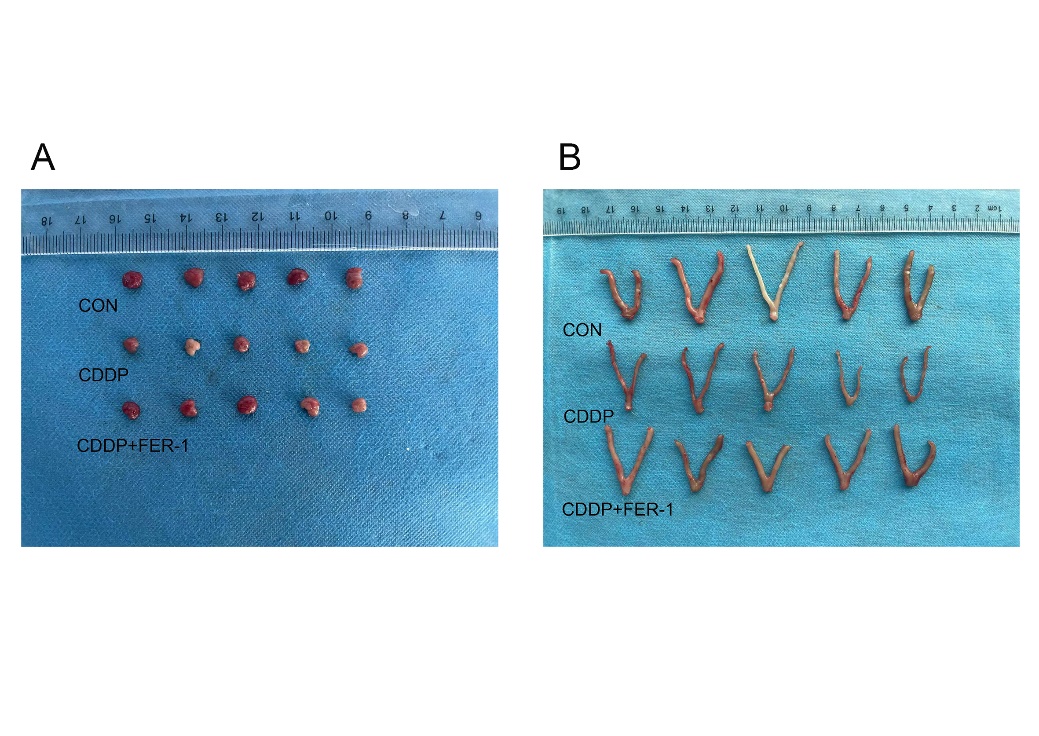


Fig.S2 The Changes in ovarian and uterine tissue during different groups. A is ovarian tissue after drug intervention in different groups; B is uterine tissue after drug intervention in different groups.

**S3 WB analysis**

A

B

C

| 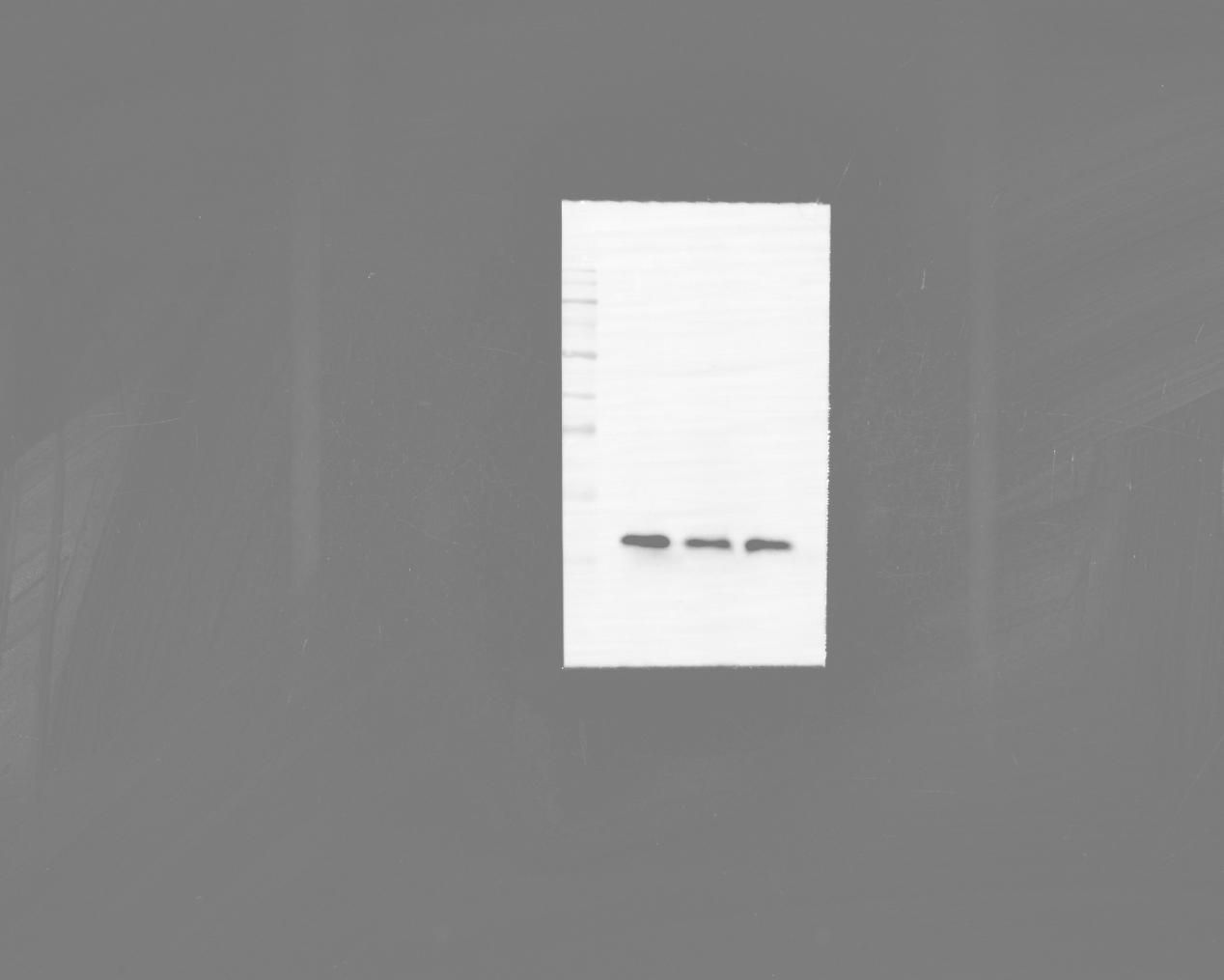 | 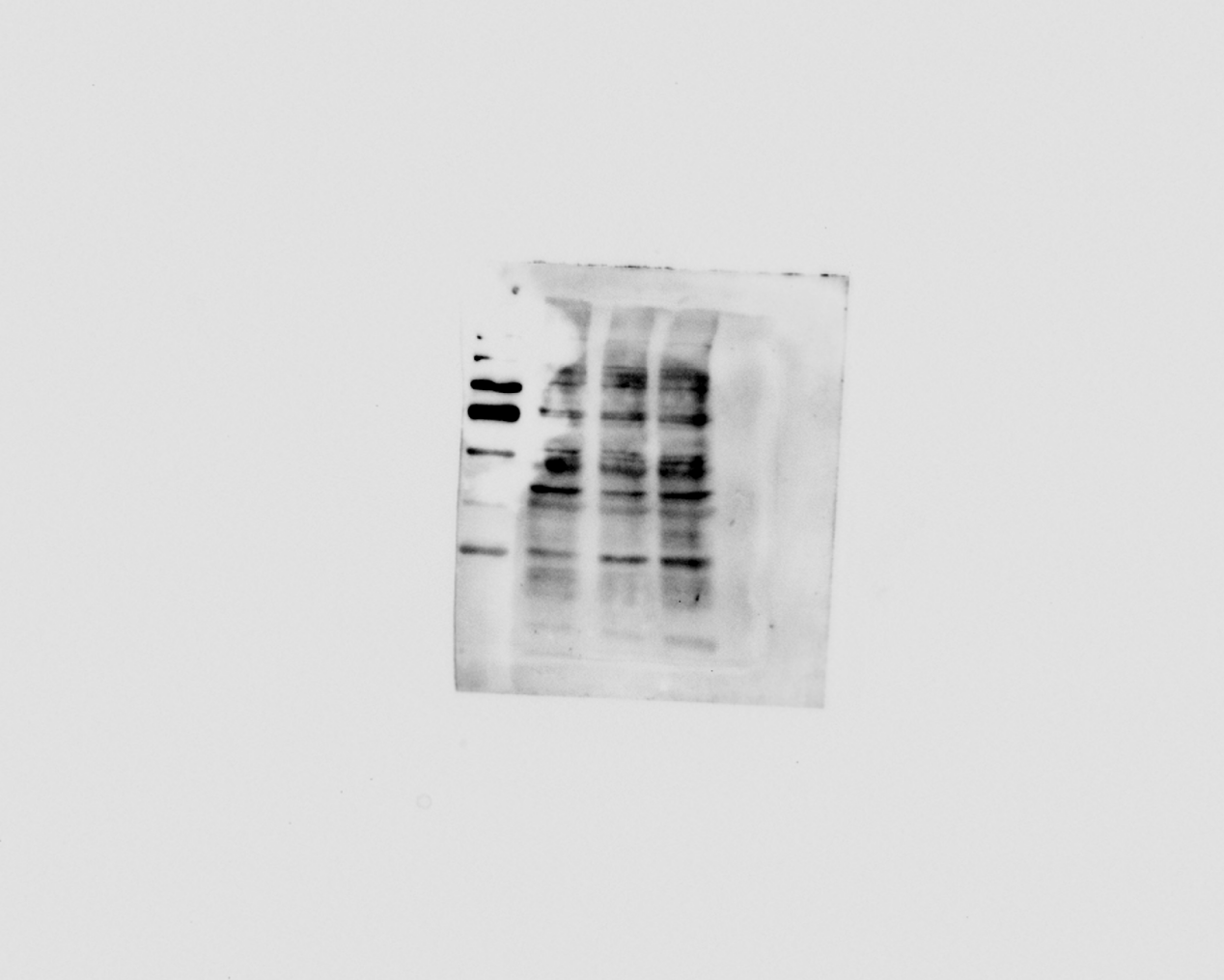 | | 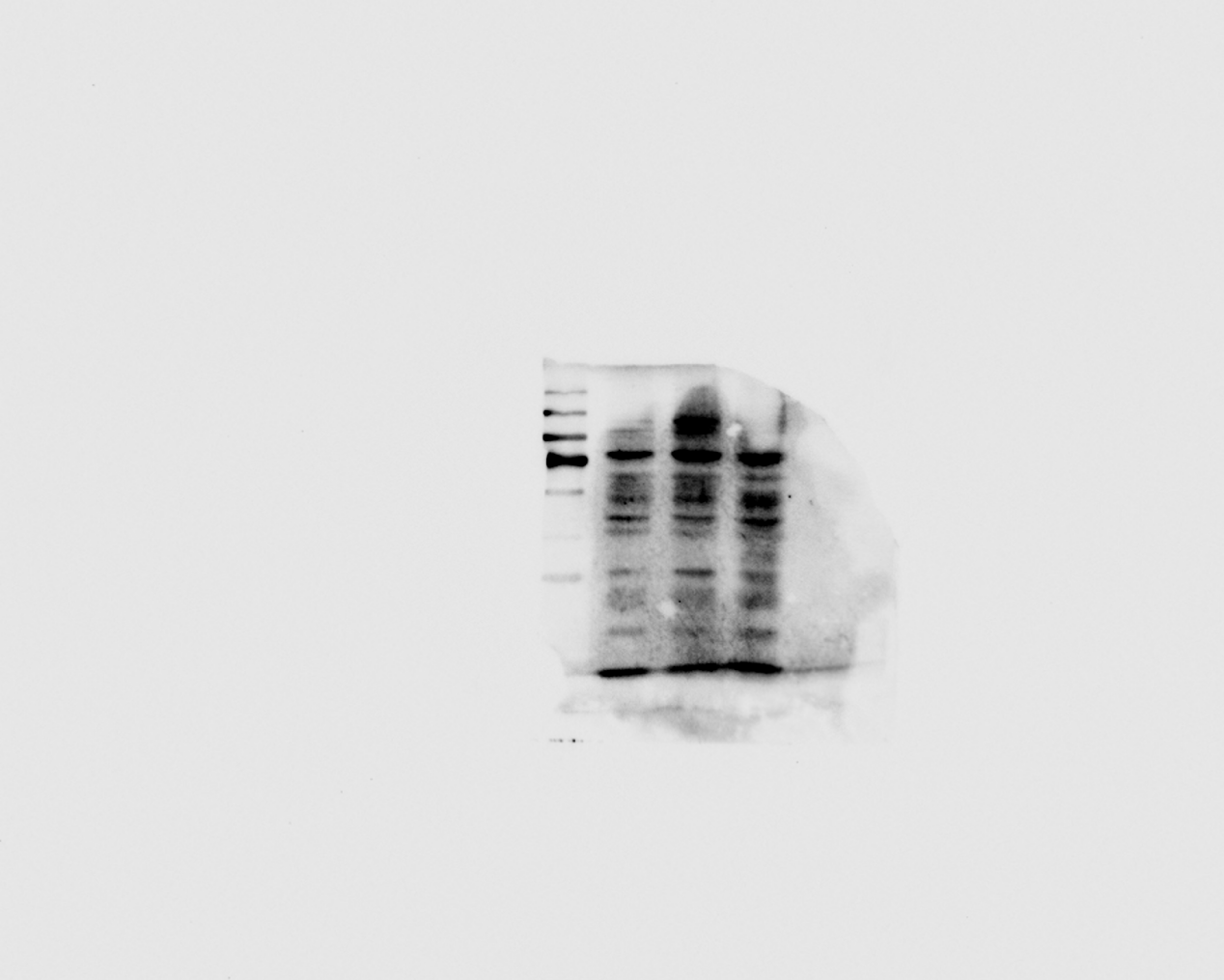 | |
| --- | --- | --- | --- | --- |
| GPX4(17kDa)  D  E | | HO-1(32kDa) | | KEAP-1(69kDa) |
| 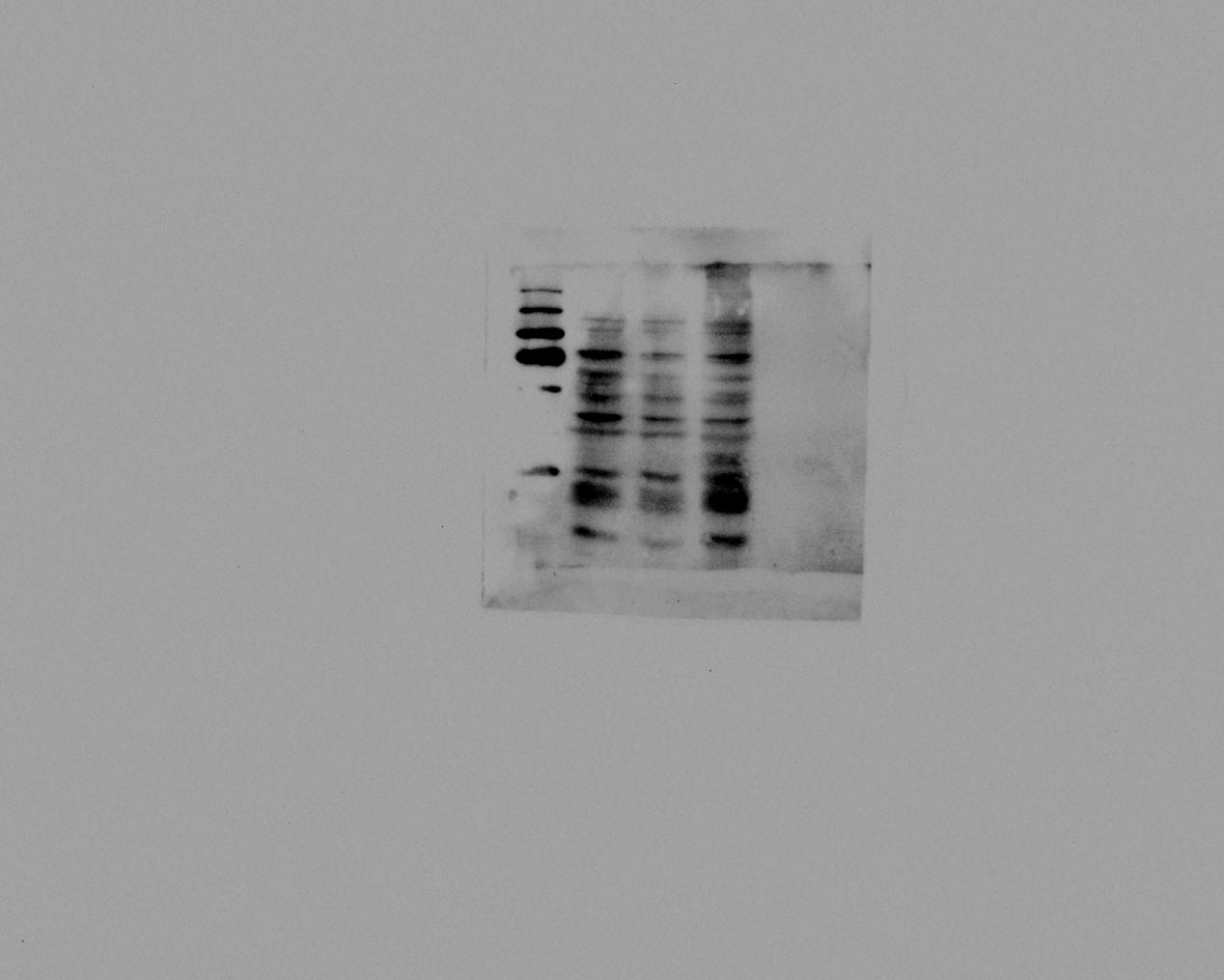 | |  | |  |
| NRF2(74kDa) | |  | |  |

Figure S3 All WB gels from figure 6 were described in detail. Marker in blue box and target protein in red box.
